# Transcatheter Versus Surgical Aortic Valve Replacement in Severe Aortic Stenosis With Reduced Left Ventricular Ejection Fraction (≤50%): A Systematic Review and Meta-Analysis of Hemodynamic and Clinical Outcomes

**DOI:** 10.7759/cureus.107896

**Published:** 2026-04-28

**Authors:** Julián Santa Cruz-Venegas, Abelardo Romero-N, Daria S. López-Rubio, A. Lucía Valenzuela-García, Brisa F. Barajas-Arciga

**Affiliations:** 1 Research, Faculty of Medicine, Universidad del Valle de México, Hermosillo, MEX; 2 Neurosciences, Universidad Tecnológica de las Islas Canarias, San Cristóbal de La Laguna, ESP; 3 General Medicine, Faculty of Medicine, Universidad del Valle de México, Hermosillo, MEX; 4 General Medicine, Faculty of Medicine, Universidad Durango Santander, Hermosillo, MEX; 5 Anaesthesiology, Faculty of Medicine, The National Autonomous University of Mexico (UNAM), Hermosillo, MEX

**Keywords:** aortic stenosis, left ventricular ejection fraction, meta-analysis, surgical aortic valve replacement, transcatheter aortic valve replacement, ventricular dysfunction

## Abstract

Severe aortic stenosis with reduced left ventricular ejection fraction (LVEF) represents a clinically vulnerable subgroup in whom the comparative benefits and risks of transcatheter aortic valve replacement/implantation (TAVR/TAVI) and surgical aortic valve replacement (SAVR) remain incompletely defined. This systematic review and meta-analysis compared early clinical outcomes and one-year hemodynamic and ventricular recovery outcomes between TAVR/TAVI and SAVR in adults with severe native aortic stenosis and baseline LVEF ≤50% or an extractable reduced-LVEF subgroup. Searches were performed in PubMed/MEDLINE, Scopus, Web of Science, Embase, and SciELO on February 27, 2026, with an updated search on April 1, 2026. Embase was used with awareness that it incorporates ClinicalTrials.gov records, and supplementary registry searches and verification included ClinicalTrials.gov, the WHO International Clinical Trials Registry Platform, and the EU Clinical Trials Register. The protocol was prospectively registered in PROSPERO (International Prospective Register of Systematic Reviews) (CRD420261348568). Risk of bias was assessed using RoB 2 (revised Cochrane risk-of-bias tool for randomized trials) for randomized evidence and ROBINS-I (Risk Of Bias In Non-randomized Studies of Interventions) V2 for nonrandomized comparative studies; certainty of evidence was evaluated using GRADE (Grading of Recommendations Assessment, Development and Evaluation). Seven comparative studies met eligibility criteria, including randomized subgroup analyses and matched or adjusted observational cohorts; six contributed to at least one pooled quantitative synthesis. Thirty-day all-cause mortality did not show a statistically significant difference between TAVR/TAVI and SAVR (RR 0.85, 95% CI 0.46-1.57; 6 studies; n=1,651). TAVR/TAVI was associated with a lower risk of early stroke (RR 0.48, 95% CI 0.25-0.91; 4 studies; n=1,285). Permanent pacemaker implantation was numerically more frequent after TAVR/TAVI, but the estimate was highly imprecise (RR 2.66, 95% CI 0.49-14.38; 3 studies; n=1,082). At one year, the pooled estimates did not establish a clear difference in mean transprosthetic gradient (MD -1.70 mmHg, 95% CI -11.22 to 7.82; 3 studies; n=654) or LVEF recovery (MD 2.91 percentage points, 95% CI -6.20 to 12.01; 3 studies; n=615). In severe aortic stenosis with reduced LVEF, TAVR/TAVI may reduce early stroke, and short-term mortality appears broadly similar to SAVR; however, evidence for pacemaker implantation, one-year gradients, and LVEF recovery remains very uncertain and should not be interpreted as evidence of equivalence between strategies.

## Introduction and background

Severe aortic stenosis (AS) is a progressive valvular disorder characterized by fixed left ventricular (LV) outflow obstruction, chronic pressure overload, concentric hypertrophy, myocardial fibrosis, and eventual ventricular decompensation. Once symptoms or LV dysfunction develop, prognosis worsens substantially without valve replacement [1]. From a guideline perspective, severe AS is generally defined using concordant echocardiographic criteria such as aortic valve area ≤1.0 cm^2^, peak aortic jet velocity ≥4.0 m/s, or mean transvalvular gradient ≥40 mmHg, while low-flow or discordant-gradient states require more nuanced evaluation [2]. Contemporary epidemiological data indicate that valvular heart disease is increasingly prevalent in aging populations, with calcific AS representing the dominant phenotype in developed settings [3].

Contemporary management of severe AS has evolved substantially with newer-generation transcatheter heart valve systems, improved delivery platforms, enhanced preprocedural imaging, and valve design modifications intended to reduce complications such as paravalvular regurgitation and vascular injury [4]. Updated ESC/EACTS (European Society of Cardiology/European Association for Cardio-Thoracic Surgery) guidance further emphasizes Heart Team decision-making, Heart Valve Centres, anatomical suitability, estimated life expectancy, procedural risk, and lifetime management when selecting between TAVR/TAVI (transcatheter aortic valve replacement/transcatheter aortic valve implantation) and SAVR [2]. The 2025 ESC/EACTS update also expanded the role of TAVI to anatomically suitable patients aged 70 years or older with tricuspid aortic valves, irrespective of estimated surgical risk, underscoring the rapidly evolving management paradigm for severe AS [2].

The broader comparative TAVR/TAVI-versus-SAVR evidence base has been shaped by major randomized trials and real-world registries across surgical-risk strata, as summarized in recent syntheses of the wider severe AS population [5]. However, these estimates were not designed around patients with impaired baseline LV systolic function and often do not center on the combination of early clinical safety, one-year valve hemodynamics, ventricular recovery, and formal certainty assessment using GRADE (Grading of Recommendations Assessment, Development and Evaluation).

Reduced baseline LVEF identifies a clinically vulnerable and pathophysiologically heterogeneous subgroup. In some patients, systolic dysfunction primarily reflects afterload mismatch, in which chronic valvular obstruction imposes excessive pressure load on the left ventricle and ventricular function may improve after relief of obstruction. In others, reduced LVEF reflects more advanced myocardial disease, including replacement fibrosis, diffuse interstitial fibrosis, ischemic injury, or intrinsic cardiomyopathy, in which ventricular recovery after valve replacement may be incomplete [1,6]. Low-flow, low-gradient presentations add further complexity because they require differentiation between true-severe AS and pseudo-severe AS, often using dobutamine stress echocardiography or complementary imaging such as CT aortic valve calcium scoring [2]. The presence or absence of contractile reserve is clinically relevant because it reflects residual myocardial functional capacity and may influence procedural risk, perioperative mortality, and the magnitude of subsequent LVEF improvement.

In this setting, residual mean transprosthetic gradient, prosthesis-patient mismatch, paravalvular regurgitation, conduction disturbances, and the trajectory of reverse remodeling may have amplified clinical consequences [7]. These issues are particularly relevant because TAVR/TAVI and SAVR differ in procedural invasiveness, use of cardiopulmonary bypass, valve platform, access route, prosthesis characteristics, risk of paravalvular regurgitation, conduction-system injury, and potential for prosthesis-patient mismatch.

Evidence focused specifically on patients with severe AS and reduced baseline LVEF remains fragmented across randomized-trial subgroup analyses, echocardiographic substudy reports, and comparative observational cohorts. Accordingly, this systematic review and meta-analysis was undertaken to compare TAVR/TAVI versus SAVR in adults with severe AS and reduced baseline LVEF (≤50% or an extractable reduced-LVEF subgroup), integrating early clinical outcomes, one-year hemodynamic and ventricular recovery endpoints, risk-of-bias assessment, and GRADE certainty ratings.

Methods

Study Design and Registration

This systematic review and meta-analysis was conducted in accordance with Preferred Reporting Items for Systematic Reviews and Meta-Analyses (PRISMA) 2020 guidance [8,9]. The protocol was prospectively registered in PROSPERO (CRD420261348568) before final data extraction and synthesis.

Search Strategy

A systematic search was performed in PubMed/MEDLINE, Scopus, Web of Science, Embase, and SciELO on February 27, 2026, with an updated search conducted on April 1, 2026. Supplementary registry verification was performed in ClinicalTrials.gov, the WHO International Clinical Trials Registry Platform (ICTRP), and the EU Clinical Trials Register; this verification did not identify additional eligible records. The strategy combined controlled vocabulary and free-text terms related to aortic stenosis, TAVR/TAVI, SAVR, ventricular function, ventricular remodeling, and comparative study designs. Searches were adapted for each database, and no publication-year restriction was applied.

Reference list screening and citation tracking were additionally undertaken to identify potentially eligible studies missed by the electronic search. The complete search strategies and the number of records retrieved from each database are provided in Table 2.

Eligibility Criteria

Studies were eligible if they enrolled adults (≥18 years) with severe native AS undergoing TAVR/TAVI or SAVR and reported outcomes for a reduced-LVEF subgroup defined as LVEF ≤50% or a directly extractable equivalent reduced-LVEF threshold reported by the study authors. Eligible designs included randomized controlled trials, randomized subgroup analyses, and comparative observational cohort studies with direct comparisons of TAVR/TAVI versus SAVR.

Comparative observational cohort studies were eligible only when they directly compared TAVR/TAVI versus SAVR in adults with severe native AS and reduced baseline LVEF and when the analysis used a matched or adjusted comparative design, such as propensity score matching, inverse probability weighting, multivariable adjustment, or another explicit method intended to address baseline confounding. Studies were also required to report sufficient baseline and outcome data to permit assessment of confounding and selection bias. Unadjusted single-arm cohorts, noncomparative reports, studies without separable reduced-LVEF outcome data, and studies comparing TAVR/TAVI with medical therapy or other nonsurgical comparators were excluded.

Conference abstracts were excluded when they lacked sufficient quantitative information for reproducible eligibility assessment or extraction, defined as the absence of treatment-group-specific denominators, event counts, or continuous outcome data, reduced-LVEF subgroup results, comparator-specific outcomes, or adequate methodological detail to assess study design and risk of bias.

Participants and Interventions

The population of interest comprised adults with severe native AS and reduced baseline LVEF. Severe AS was accepted according to the definition used by each included study, typically based on aortic valve area, Doppler gradients, peak aortic jet velocity, or guideline-concordant composite criteria. This approach was necessary because the included studies did not consistently report standardized diagnostic thresholds, dobutamine stress echocardiography findings, CT calcium scoring, flow-gradient phenotype, or contractile reserve.

Severe AS in the setting of reduced LVEF is diagnostically heterogeneous. Patients with high-gradient severe AS may differ substantially from those with classical low-flow, low-gradient AS, in whom differentiation between true-severe and pseudo-severe stenosis is essential. The presence or absence of contractile reserve may also influence procedural risk, perioperative mortality, and the likelihood of LVEF recovery after valve replacement. Therefore, the pooled estimates should be interpreted as applying to a broad reduced-LVEF severe AS population rather than to a specific flow-gradient phenotype.

Interventions were categorized at the treatment-strategy level as TAVR/TAVI versus SAVR. TAVR/TAVI may involve balloon-expandable or self-expanding valves, different device generations, and transfemoral or nontransfemoral access routes. SAVR may involve conventional sternotomy or minimally invasive approaches and different prosthesis types. Because the included reduced-LVEF studies did not consistently report outcome data stratified by valve platform, access route, surgical approach, or prosthesis type, subgroup analyses by procedural subtype were not feasible. Therefore, the pooled estimates compare broad treatment strategies rather than device-specific or technique-specific effects.

Outcomes

Primary outcomes: The prespecified primary clinical outcome was 30-day all-cause mortality, interpreted as an early procedural safety endpoint rather than a comprehensive measure of longer-term disease progression. Cardiovascular mortality, heart failure rehospitalization, and composite mortality/heart failure endpoints were considered clinically relevant but were not consistently reported in extractable form for the reduced-LVEF subgroup across included studies; therefore, they could not be pooled reliably without introducing additional assumptions or selective outcome inclusion.

The prespecified primary hemodynamic outcome was one-year follow-up mean transprosthetic gradient, or the closest available follow-up around one year when exact timing varied slightly across studies. This variable was extracted as the study-reported follow-up Doppler echocardiographic mean gradient when available. Gradients were not flow-adjusted because the included studies did not consistently provide subgroup-level data on stroke volume index, low-flow status, contractile reserve, invasive hemodynamic measurements, or longitudinal flow recovery.

Secondary outcomes: Secondary outcomes were early stroke (30-day or in-hospital/periprocedural when directly comparable to the early postprocedural window), permanent pacemaker implantation, and one-year follow-up LVEF.

Study Selection and Data Extraction

Two reviewers independently screened titles and abstracts and subsequently assessed potentially eligible full texts. Disagreements were resolved by consensus. The study selection process was documented using a PRISMA 2020 flow diagram [8,9]. Data extraction was performed in duplicate using a standardized form. Extracted items included study design, country or setting, sample size analyzed, age, female sex distribution, operative risk score, atrial fibrillation prevalence, reduced-LVEF definition, severe AS criteria when available, device or access-route information when reported, echocardiographic follow-up timing, and numerical outcome data for dichotomous and continuous variables.

When multiple reports described the same trial, registry, or parent cohort, reports were collated at the parent-study level rather than treated as independent studies. For each outcome-specific meta-analysis, only one nonoverlapping dataset per study population was included. When overlapping reports provided data for the same outcome and time point, we preferentially extracted the most complete and directly relevant reduced-LVEF subgroup data. Additional reports were used only to supplement missing baseline characteristics, follow-up information, risk-of-bias assessment, or outcomes not available in the primary report. Duplicate event counts, means, standard deviations, sample sizes, or overlapping patient groups were not entered more than once into the same pooled analysis.

Mean transprosthetic gradient and LVEF were extracted from study-reported echocardiographic follow-up data, generally based on transthoracic echocardiography when specified. When available, we recorded the timing of echocardiographic follow-up and whether the study reported standardized image acquisition, blinded interpretation, or core-laboratory adjudication. However, the included studies did not consistently report echocardiographic quality-control procedures, Doppler acquisition protocols, core-laboratory assessment, or blinding of echocardiographic interpretation.

Risk of Bias Assessment

Risk of bias was assessed according to the study design. RoB 2 was applied to randomized evidence, and ROBINS-I V2 was applied to non-randomized comparative follow-up cohort studies [10,11]. For observational evidence, residual confounding and treatment-selection bias were considered central domains, particularly because surgical risk, frailty, porcelain aorta, coronary disease burden, anatomical suitability, STS-PROM, EuroSCORE, and other factors may influence the selection of TAVR/TAVI versus SAVR. Domain-level judgments were used to derive overall outcome-level interpretation.

Data Synthesis and Statistical Analysis

Dichotomous outcomes were synthesized as pooled risk ratios (RRs) with 95% confidence intervals (CIs), and continuous outcomes as pooled mean differences (MDs) with 95% CIs. Random-effects models were used a priori because clinical and methodological heterogeneity were expected across the included studies. Dichotomous and continuous outcomes were pooled using the inverse-variance method. Between-study variance (τ2) was estimated using the Paule-Mandel estimator, and confidence intervals around pooled random-effects estimates were calculated using the Hartung-Knapp adjustment. Prediction intervals were calculated for pooled random-effects analyses to reflect the expected dispersion of effects across settings.

Statistical heterogeneity was assessed using I2, τ2, and Cochran’s Q test. Meta-analysis was performed only when at least two studies reported sufficiently comparable data for a given outcome. Direction of effect was interpreted relative to TAVR/TAVI versus SAVR. Because fewer than 10 studies contributed to each outcome-specific synthesis, formal funnel plot asymmetry testing and Egger’s or Begg’s tests were not performed. All quantitative analyses and forest plots were performed in R version 4.4.2 (R Foundation for Statistical Computing, Vienna, Austria) within RStudio version 2026.01.1+403 (Posit Software, PBC, Boston, MA, USA), using the meta package version 8.2-1.

Formal subgroup analyses by LVEF severity, study era, valve platform, access route, surgical approach, low-flow status, contractile reserve, or echocardiographic quality were not performed because the number of studies contributing to each outcome was very small and because the included studies did not consistently report stratified outcome data by these variables. Under these conditions, post hoc subgroup analyses would have been statistically unstable and potentially misleading.

Assessment of Certainty of the Evidence

The certainty of the evidence was assessed using the GRADE approach for each pooled outcome, considering risk of bias, inconsistency, indirectness, imprecision, and publication bias [12,13]. Certainty was categorized as high, moderate, low, or very low. GRADE judgments incorporated risk of bias related to randomized subgroup evidence and residual confounding in observational comparisons, as well as imprecision, inconsistency, and the limited ability to assess publication bias in sparse meta-analyses. Detailed certainty assessments are provided in Tables 4, 5.

## Review

Results

Study Selection and Characteristics

**Figure 1:**
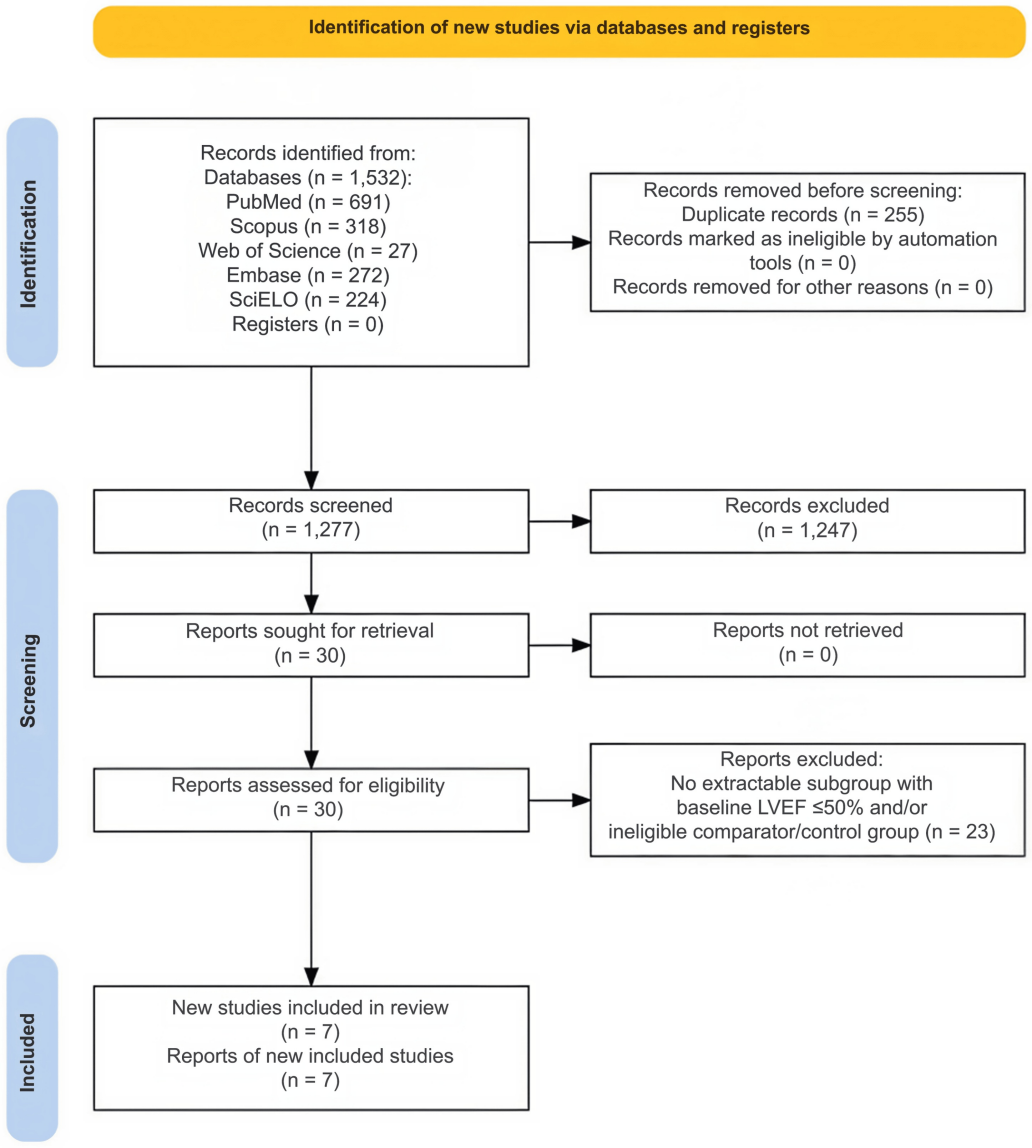
PRISMA 2020 flow diagram of study identification, screening, eligibility assessment, and inclusion. PRISMA: Preferred Reporting Items for Systematic Reviews and Meta-Analyses.

The search identified 1,532 records across databases and the updated search. After removing 255 duplicate records, 1,277 records underwent title and abstract screening. The large reduction from screened records to full-text assessment reflected the narrow eligibility framework of this review. Records were excluded at the title/abstract stage when they clearly did not include adults with severe native AS, did not evaluate a reduced-LVEF subgroup, did not directly compare TAVR/TAVI versus SAVR, were non-comparative, or did not report extractable clinical or hemodynamic outcomes relevant to the review question. Thirty reports were assessed in full text, and 23 were excluded. Seven studies were ultimately included in the systematic review; six contributed to at least one pooled quantitative synthesis, whereas Ito et al. contributed comparative prognostic data to the qualitative synthesis and risk-of-bias assessment only (Figure 1).

**Table 1:** Characteristics of included studies. AS, aortic stenosis; AVR, aortic valve replacement; EF, ejection fraction; LVEF, left ventricular ejection fraction; TAVR, transcatheter aortic valve replacement; TAVI, transcatheter aortic valve implantation; SAVR, surgical aortic valve replacement; SVI, stroke volume index; SD, standard deviation; IQR, interquartile range; STS, Society of Thoracic Surgeons Predicted Risk of Mortality score; EuroSCORE, European System for Cardiac Operative Risk Evaluation; NR, not reported.

Study	Sample size analyzed	Age, years	Female sex, n/N (%)	Risk score	Atrial fibrillation	Reduced-LVEF population	Main outcomes contributed
Clavel et al., 2010 [14]	283 total (TAVI 83; SAVR 200)	TAVR 81; SAVR 70	TAVR 34/83 (41.0%); SAVR 38/200 (19.0%)	Logistic EuroSCORE: TAVR 32 ± 18; SAVR 18 ± 14; STS: TAVR 12 ± 7; SAVR 6 ± 5	TAVR 32 (39%); SAVR 46 (23%)	Severe AS with LVEF ≤50%	30-day mortality; 1-year mean transprosthetic gradient
Elmariah et al., 2013 [15]	LVEF <50% subgroup: TAVR 108; SAVR 95	TAVR 83; SAVR 84	TAVR 34/108 (31.5%); SAVR 31/95 (32.6%)	STS score: TAVR 12.2 ± 3.7; SAVR 12.0 ± 2.9	TAVR 55 (50.9%); SAVR 48 (50.5%)	High-risk severe AS with LVEF <50%	30-day mortality; early stroke; 1-year LVEF
Onorati et al., 2014 [16]	162 matched patients (TAVI 81; AVR 81)	TAVR 77.5; SAVR 77.6	TAVR 27/81 (33.3%); SAVR 31/81 (38.3%)	Logistic EuroSCORE: TAVR 21.4 ± 14.3; SAVR 25.9 ± 18.8	NR	Severe AS with LVEF ≤35%	30-day mortality; early stroke; permanent pacemaker implantation
Ito et al., 2020 [17]	Baseline SVI/LVEF available: TAVR 348; SAVR 306	TAVR 82.7; SAVR 82.9	TAVR 48/125 (38.4%); SAVR 36/96 (37.5%)	Logistic EuroSCORE: TAVR 20.9 ± 15.7; SAVR 20.6 ± 14.9	TAVR 69 (55.6%); SAVR 53 (55.2%)	High-risk severe AS stratified by LVEF <50%	Comparative prognostic data; contributed to qualitative synthesis and risk-of-bias assessment
Jalava et al., 2022 [18]	255 matched pairs (TAVR 255; SAVR 255)	TAVR 79.2; SAVR 79.8	TAVR 106/255 (41.6%); SAVR 111/255 (43.5%)	EuroSCORE II: TAVR 9.3 ± 8.9; SAVR 8.7 ± 7.9	TAVR 113 (44.3%); SAVR 117 (45.9%)	Severe AS with LVEF ≤50%	30-day mortality; early stroke; permanent pacemaker implantation; baseline hemodynamics
Bain et al., 2024 [19]	83 total (TAVR 56; SAVR 27)	73.4	TAVR 15/56 (26.8%); SAVR 8/27 (29.6%)	NR	TAVR 18 (32.1%); SAVR 7 (25.9%)	Severe AS with LVEF ≤25%	30-day mortality; 1-year/closest follow-up gradient and LVEF; baseline hemodynamics
Brown et al., 2026 [20]	205 matched pairs (TAVR 205; SAVR 205)	TAVR 74 [68–80]; SAVR 73 [68–80]	TAVR 51/205 (24.9%); SAVR 53/205 (25.9%)	STS mortality risk: TAVR 3.9 [2.4–6.0]; SAVR 2.7 [1.6–5.5]	NR	Severe AS with reduced EF <50%	30-day mortality; early stroke; permanent pacemaker implantation; 1-year gradient and LVEF

Characteristics of the Included Studies

The seven included studies comprised two randomized subgroup analyses and five comparative observational cohorts [14-20]. The evidence base included one multicenter observational comparative study in patients with LVEF ≤50% (Clavel et al., 2010) [14], one PARTNER Cohort A randomized subgroup analysis in high-risk severe AS with LVEF <50% (Elmariah et al., 2013) [15], one propensity-matched registry-based cohort in patients with LVEF ≤35% (Onorati et al., 2014) [16], one high-risk CoreValve randomized subgroup analysis stratified by LVEF <50% that contributed qualitative prognostic data only (Ito et al., 2020) [17], one nationwide propensity-matched cohort in patients with LVEF ≤50% (Jalava et al., 2022) [18], one single-center retrospective cohort restricted to LVEF ≤25% (Bain et al., 2024) [19], and one recent propensity-matched retrospective cohort in patients with reduced EF <50% (Brown et al., 2026) [20].

The included studies were clinically heterogeneous with respect to design, sample size, baseline operative risk, procedural era, and LVEF threshold. Earlier cohorts tended to include higher-risk TAVR/TAVI patients and reflect less contemporary device generations and procedural protocols, whereas later matched cohorts may better approximate current practice. Baseline operative risk was reported using different instruments. Clavel et al. reported higher Logistic EuroSCORE and STS values in the TAVR/TAVI group than in the SAVR group, Elmariah et al. reported similar STS scores, Onorati et al. reported comparable Logistic EuroSCORE values after matching, Ito et al. reported similar Logistic EuroSCORE values, Jalava et al. reported similar EuroSCORE II values, and Brown et al. reported STS mortality risk values for both groups. Bain et al. did not report a formal surgical risk score. These differences were compared descriptively rather than quantitatively.

Outcome availability varied across studies. Although seven studies were included in the systematic review, fewer studies contributed to each outcome-specific meta-analysis. In particular, the one-year mean transprosthetic gradient and one-year LVEF analyses were each based on only three studies, representing a smaller subset of the total evidence base. This fragmented outcome reporting and the broad range of LVEF thresholds should be considered when interpreting the pooled estimates, particularly for one-year hemodynamic and ventricular recovery outcomes. Table 1 summarizes the main characteristics and outcomes contributed by each included study. 

**Table 2:** Search strategy on each database.

Engine	Search date: February 27, 2026, with an updated search conducted on April 1, 2026.	Results
Pubmed	("Aortic Valve Disease" OR "Aortic Valve Diseases" OR "Valve Disease, Aortic" OR "Aortic Valvular Heart Disease" OR "Aortic Valve Disorder" OR "Aortic Valve Disorders" OR "Valve Disorder, Aortic" OR "Aortic Valvular Heart Disorder" OR "Aortic Heart Disease" OR "Aortic Heart Diseases" OR "Heart Disease, Aortic" OR "Aortic Valve Stenoses" OR "Stenoses, Aortic Valve" OR "Stenosis, Aortic Valve" OR "Valve Stenoses, Aortic" OR "Valve Stenosis, Aortic" OR "Aortic Stenosis" OR "Stenoses, Aortic" OR "Stenosis, Aortic")	691
	("TAVR" OR "Transcatheter Aortic Valve Implantation" OR "Implantation, Heart Valve Prosthesis" OR "Surgical Procedure, Cardiac" OR "Surgical Procedures, Cardiac" OR "Heart Surgical Procedure" OR "Heart Surgical Procedures" OR "Procedure, Heart Surgical" OR "Procedures, Heart Surgical" OR "Surgical Procedure, Heart" OR "Surgical Procedures, Heart" OR "Cardiac Surgical Procedure" OR "Procedures, Cardiac Surgical" OR "Procedure, Cardiac Surgical")	
	("Ventricular Function, Left" OR "Left Ventricular Function" OR "Function, Left Ventricular" OR "Functions, Left Ventricular" OR "Left Ventricular Functions" OR "Ventricular Functions, Left" OR "Ventricular Remodeling" OR "Remodelings, Ventricular" OR "Remodeling, Ventricular" OR "Ventricular Remodelings" OR "Cardiac Remodeling, Ventricular" OR "Cardiac Remodelings, Ventricular" OR "Remodelings, Ventricular Cardiac" OR "Remodeling, Ventricular Cardiac" OR "Ventricular Cardiac Remodeling" OR "Ventricular Cardiac Remodelings" OR "Myocardial Remodeling, Ventricular" OR "Myocardial Remodelings, Ventricular" OR "Remodelings, Ventricular Myocardial" OR "Remodeling, Ventricular Myocardial" OR "Ventricular Myocardial Remodeling" OR "Ventricular Myocardial Remodelings" OR "Ventricle Remodeling" OR "Remodelings, Ventricle" OR "Remodeling, Ventricle" OR "Ventricle Remodelings" OR "Left Ventricle Remodeling" OR "Left Ventricle Remodelings" OR "Remodeling, Left Ventricle" OR "Remodelings, Left Ventricle" OR "Ventricle Remodeling, Left" OR "Ventricle Remodelings, Left" OR "Left Ventricular Remodeling" OR "Left Ventricular Remodelings" OR "Remodeling, Left Ventricular" OR "Remodelings, Left Ventricular" OR "Ventricular Remodeling, Left" OR "Ventricular Remodelings, Left" OR "Ventricular Pressure" OR "Pressures, Ventricular" OR "Pressure, Ventricular" OR "Ventricular Pressures" OR "Intraventricular Pressure" OR "Intraventricular Pressures" OR "Pressure, Intraventricular" OR "Pressures, Intraventricular" OR "Stroke Volumes" OR "Volumes, Stroke" OR "Volume, Stroke" OR "Ventricular Ejection Fraction" OR "Ejection Fractions, Ventricular" OR "Ejection Fraction, Ventricular" OR "Fractions, Ventricular Ejection" OR "Fraction, Ventricular Ejection" OR "Ventricular Ejection Fractions" OR "Ventricular End-Diastolic Volume" OR "End-Diastolic Volumes, Ventricular" OR "End-Diastolic Volume, Ventricular" OR "Ventricular End Diastolic Volume" OR "Ventricular End-Diastolic Volumes" OR "Volumes, Ventricular End-Diastolic" OR "Volume, Ventricular End-Diastolic" OR "Ventricular End-Systolic Volume" OR "End-Systolic Volumes, Ventricular" OR "End-Systolic Volume, Ventricular" OR "Ventricular End Systolic Volume" OR "Ventricular End-Systolic Volumes" OR "Volumes, Ventricular End-Systolic" OR "Volume, Ventricular End-Systolic")	
	(“Randomized Controlled Trial” OR “Randomised Controlled Trial” OR “Clinical Trials, Randomized" OR “Trials, Randomized Clinical” OR “Controlled Clinical Trials, Randomized” OR “controlled clinical trial” OR “clinical trial”)	
Scopus	TITLE-ABS-KEY ("Aortic Valve Disease" OR "Aortic Valve Diseases" OR "Valve Disease, Aortic" OR "Aortic Valvular Heart Disease" OR "Aortic Valve Disorder" OR "Aortic Valve Disorders" OR "Valve Disorder, Aortic" OR "Aortic Valvular Heart Disorder" OR "Aortic Heart Disease" OR "Aortic Heart Diseases" OR "Heart Disease, Aortic" OR "Aortic Valve Stenoses" OR "Stenoses, Aortic Valve" OR "Stenosis, Aortic Valve" OR "Valve Stenoses, Aortic" OR "Valve Stenosis, Aortic" OR "Aortic Stenosis" OR "Stenoses, Aortic" OR "Stenosis, Aortic")	318
	TITLE-ABS-KEY ("TAVR" OR "Transcatheter Aortic Valve Implantation" OR "Implantation, Heart Valve Prosthesis" OR "Surgical Procedure, Cardiac" OR "Surgical Procedures, Cardiac" OR "Heart Surgical Procedure" OR "Heart Surgical Procedures" OR "Procedure, Heart Surgical" OR "Procedures, Heart Surgical" OR "Surgical Procedure, Heart" OR "Surgical Procedures, Heart" OR "Cardiac Surgical Procedure" OR "Procedures, Cardiac Surgical" OR "Procedure, Cardiac Surgical")	
	TITLE-ABS-KEY ("Ventricular Function, Left" OR "Left Ventricular Function" OR "Function, Left Ventricular" OR "Functions, Left Ventricular" OR "Left Ventricular Functions" OR "Ventricular Functions, Left" OR "Ventricular Remodeling" OR "Remodelings, Ventricular" OR "Remodeling, Ventricular" OR "Ventricular Remodelings" OR "Cardiac Remodeling, Ventricular" OR "Cardiac Remodelings, Ventricular" OR "Remodelings, Ventricular Cardiac" OR "Remodeling, Ventricular Cardiac" OR "Ventricular Cardiac Remodeling" OR "Ventricular Cardiac Remodelings" OR "Myocardial Remodeling, Ventricular" OR "Myocardial Remodelings, Ventricular" OR "Remodelings, Ventricular Myocardial" OR "Remodeling, Ventricular Myocardial" OR "Ventricular Myocardial Remodeling" OR "Ventricular Myocardial Remodelings" OR "Ventricle Remodeling" OR "Remodelings, Ventricle" OR "Remodeling, Ventricle" OR "Ventricle Remodelings" OR "Left Ventricle Remodeling" OR "Left Ventricle Remodelings" OR "Remodeling, Left Ventricle" OR "Remodelings, Left Ventricle" OR "Ventricle Remodeling, Left" OR "Ventricle Remodelings, Left" OR "Left Ventricular Remodeling" OR "Left Ventricular Remodelings" OR "Remodeling, Left Ventricular" OR "Remodelings, Left Ventricular" OR "Ventricular Remodeling, Left" OR "Ventricular Remodelings, Left" OR "Ventricular Pressure" OR "Pressures, Ventricular" OR "Pressure, Ventricular" OR "Ventricular Pressures" OR "Intraventricular Pressure" OR "Intraventricular Pressures" OR "Pressure, Intraventricular" OR "Pressures, Intraventricular" OR "Stroke Volumes" OR "Volumes, Stroke" OR "Volume, Stroke" OR "Ventricular Ejection Fraction" OR "Ejection Fractions, Ventricular" OR "Ejection Fraction, Ventricular" OR "Fractions, Ventricular Ejection" OR "Fraction, Ventricular Ejection" OR "Ventricular Ejection Fractions" OR "Ventricular End-Diastolic Volume" OR "End-Diastolic Volumes, Ventricular" OR "End-Diastolic Volume, Ventricular" OR "Ventricular End Diastolic Volume" OR "Ventricular End-Diastolic Volumes" OR "Volumes, Ventricular End-Diastolic" OR "Volume, Ventricular End-Diastolic" OR "Ventricular End-Systolic Volume" OR "End-Systolic Volumes, Ventricular" OR "End-Systolic Volume, Ventricular" OR "Ventricular End Systolic Volume" OR "Ventricular End-Systolic Volumes" OR "Volumes, Ventricular End-Systolic" OR "Volume, Ventricular End-Systolic")	
	TITLE-ABS-KEY(“Randomized Controlled Trial” OR “Randomised Controlled Trial” OR “Clinical Trials, Randomized" OR “Trials, Randomized Clinical” OR “Controlled Clinical Trials, Randomized” OR “controlled clinical trial” OR “clinical trial”)	
Web of Science	("Aortic Valve Disease" OR "Aortic Valve Diseases" OR "Valve Disease, Aortic" OR "Aortic Valvular Heart Disease" OR "Aortic Valve Disorder" OR "Aortic Valve Disorders" OR "Valve Disorder, Aortic" OR "Aortic Valvular Heart Disorder" OR "Aortic Heart Disease" OR "Aortic Heart Diseases" OR "Heart Disease, Aortic" OR "Aortic Valve Stenoses" OR "Stenoses, Aortic Valve" OR "Stenosis, Aortic Valve" OR "Valve Stenoses, Aortic" OR "Valve Stenosis, Aortic" OR "Aortic Stenosis" OR "Stenoses, Aortic" OR "Stenosis, Aortic")	27
	("TAVR" OR "Transcatheter Aortic Valve Implantation" OR "Implantation, Heart Valve Prosthesis" OR "Surgical Procedure, Cardiac" OR "Surgical Procedures, Cardiac" OR "Heart Surgical Procedure" OR "Heart Surgical Procedures" OR "Procedure, Heart Surgical" OR "Procedures, Heart Surgical" OR "Surgical Procedure, Heart" OR "Surgical Procedures, Heart" OR "Cardiac Surgical Procedure" OR "Procedures, Cardiac Surgical" OR "Procedure, Cardiac Surgical")	
	("Ventricular Function, Left" OR "Left Ventricular Function" OR "Function, Left Ventricular" OR "Functions, Left Ventricular" OR "Left Ventricular Functions" OR "Ventricular Functions, Left" OR "Ventricular Remodeling" OR "Remodelings, Ventricular" OR "Remodeling, Ventricular" OR "Ventricular Remodelings" OR "Cardiac Remodeling, Ventricular" OR "Cardiac Remodelings, Ventricular" OR "Remodelings, Ventricular Cardiac" OR "Remodeling, Ventricular Cardiac" OR "Ventricular Cardiac Remodeling" OR "Ventricular Cardiac Remodelings" OR "Myocardial Remodeling, Ventricular" OR "Myocardial Remodelings, Ventricular" OR "Remodelings, Ventricular Myocardial" OR "Remodeling, Ventricular Myocardial" OR "Ventricular Myocardial Remodeling" OR "Ventricular Myocardial Remodelings" OR "Ventricle Remodeling" OR "Remodelings, Ventricle" OR "Remodeling, Ventricle" OR "Ventricle Remodelings" OR "Left Ventricle Remodeling" OR "Left Ventricle Remodelings" OR "Remodeling, Left Ventricle" OR "Remodelings, Left Ventricle" OR "Ventricle Remodeling, Left" OR "Ventricle Remodelings, Left" OR "Left Ventricular Remodeling" OR "Left Ventricular Remodelings" OR "Remodeling, Left Ventricular" OR "Remodelings, Left Ventricular" OR "Ventricular Remodeling, Left" OR "Ventricular Remodelings, Left" OR "Ventricular Pressure" OR "Pressures, Ventricular" OR "Pressure, Ventricular" OR "Ventricular Pressures" OR "Intraventricular Pressure" OR "Intraventricular Pressures" OR "Pressure, Intraventricular" OR "Pressures, Intraventricular" OR "Stroke Volumes" OR "Volumes, Stroke" OR "Volume, Stroke" OR "Ventricular Ejection Fraction" OR "Ejection Fractions, Ventricular" OR "Ejection Fraction, Ventricular" OR "Fractions, Ventricular Ejection" OR "Fraction, Ventricular Ejection" OR "Ventricular Ejection Fractions" OR "Ventricular End-Diastolic Volume" OR "End-Diastolic Volumes, Ventricular" OR "End-Diastolic Volume, Ventricular" OR "Ventricular End Diastolic Volume" OR "Ventricular End-Diastolic Volumes" OR "Volumes, Ventricular End-Diastolic" OR "Volume, Ventricular End-Diastolic" OR "Ventricular End-Systolic Volume" OR "End-Systolic Volumes, Ventricular" OR "End-Systolic Volume, Ventricular" OR "Ventricular End Systolic Volume" OR "Ventricular End-Systolic Volumes" OR "Volumes, Ventricular End-Systolic" OR "Volume, Ventricular End-Systolic")	
	(“Randomized Controlled Trial” OR “Randomised Controlled Trial” OR “Clinical Trials, Randomized" OR “Trials, Randomized Clinical” OR “Controlled Clinical Trials, Randomized” OR “controlled clinical trial” OR “clinical trial”)	
SciELO Brazil (DeCS/MeSH multilingual strategy: English, Spanish, Portuguese, and French)	("Aortic Valve Stenosis" OR "Aortic Stenosis" OR "Aortic Valve Stenoses" OR "Stenoses, Aortic" OR "Stenoses, Aortic Valve" OR "Stenosis, Aortic" OR "Stenosis, Aortic Valve" OR "Valve Stenoses, Aortic" OR "Valve Stenosis, Aortic" OR "Estenosis de la Válvula Aórtica" OR "Estenosis Aórtica" OR "Estenose da Valva Aórtica" OR "Estenose Aórtica" OR "Sténose aortique" OR "Rétrécissement aortique" OR "Rétrécissement aortique valvulaire" OR "Rétrecissement valvulaire aortique" OR "Sténose aortique valvulaire" OR "Sténose de la valve aortique" OR "Sténose valvulaire aortique" OR "Aortic Valve Disease" OR "Aortic Heart Disease" OR "Aortic Heart Diseases" OR "Aortic Valve Diseases" OR "Aortic Valve Disorder" OR "Aortic Valve Disorders" OR "Aortic Valvular Heart Disease" OR "Aortic Valvular Heart Disorder" OR "Heart Disease, Aortic" OR "Valve Disease, Aortic" OR "Valve Disorder, Aortic" OR "Enfermedad de la Válvula Aórtica" OR "Enfermedad Cardíaca Aórtica" OR "Enfermedad Valvular Aórtica del Corazón" OR "Trastorno de la Válvula Aórtica" OR "Trastorno Valvular Aórtica del Corazón" OR "Valvulopatía Aórtica" OR "Valvopatia Aórtica" OR "Doença da Valva Aórtica" OR "Doença da Válvula Aórtica" OR "Doenças da Valva Aórtica" OR "Doenças da Válvula Aórtica" OR "Transtorno da Válvula Aórtica" OR "Transtorno Valvular Aórtico do Coração" OR "Valvopatia Aórtica Cardíaca" OR "Valvopatias Aórticas" OR "Maladie de la valve aortique" OR "Cardiopathie aortique" OR "Cardiopathie valvulaire aortique" OR "Maladie cardiaque aortique" OR "Trouble cardiaque valvulaire aortique" OR "Trouble de la valve aortique" OR "Valvulopathie aortique")	224
	("Cardiac Surgical Procedures" OR "Cardiac Surgical Procedure" OR "Heart Surgical Procedure" OR "Heart Surgical Procedures" OR "Procedure, Cardiac Surgical" OR "Procedure, Heart Surgical" OR "Procedures, Cardiac Surgical" OR "Procedures, Heart Surgical" OR "Surgical Procedure, Cardiac" OR "Surgical Procedure, Heart" OR "Surgical Procedures, Cardiac" OR "Surgical Procedures, Heart" OR "Procedimientos Quirúrgicos Cardíacos" OR "Procedimentos Cirúrgicos Cardíacos" OR "Procédures de chirurgie cardiaque" OR "Interventions chirurgicales cardiaques" OR "Interventions de cardiochirurgie" OR "Interventions de chirurgie cardiaque" OR "Procédures chirurgicales cardiaques" OR "Procédures de cardiochirurgie" OR "Transcatheter Aortic Valve Replacement" OR "TAVR" OR "Transcatheter Aortic Valve Implantation" OR "Reemplazo de la Válvula Aórtica Transcatéter" OR "Implante Transcatéter de Prótesis Valvular Aórtica" OR "Substituição da Valva Aórtica Transcateter" OR "Remplacement valvulaire aortique par cathéter" OR "Implantation de valve aortique par cathéter" OR "Implantation de valvule aortique par cathéter" OR "Implantation valvulaire aortique par cathéter" OR "IVAC" OR "Remplacement de la valve aortique par cathéter" OR "Remplacement de la valvule aortique par cathéter" OR "Remplacement de valve aortique par cathéter" OR "Remplacement de valvule aortique par cathéter" OR "RVAC" OR "Heart Valve Prosthesis Implantation" OR "Implantation, Heart Valve Prosthesis" OR "Implantación de Prótesis de Válvulas Cardíacas" OR "Implante de Prótese de Valva Cardíaca" OR "Implantação de Prótese de Valva" OR "Implantação de Prótese de Valva Cardíaca" OR "Implantação de Prótese Valvar Cardíaca" OR "Implante de Prótese de Valva" OR "Implante de Prótese Valvar Cardíaca" OR "Implantation de valve prothétique cardiaque" OR "Implantation de prothèse de valve cardiaque" OR "Implantation de prothèse valvaire cardiaque" OR "Implantation de prothèse valvulaire cardiaque" OR "Pose de prothèse valvulaire cardiaque" OR "Remplacement de valve cardiaque" OR "Remplacement des valve cardiaques"OR "Remplacement valvaire cardiaque" OR "Remplacement valvulaire cardiaque")	
	("Stroke Volume" OR "Ejection Fraction, Ventricular" OR "Ejection Fractions, Ventricular" OR "End-Diastolic Volume, Ventricular" OR "End-Diastolic Volumes, Ventricular" OR "End-Systolic Volume, Ventricular" OR "End-Systolic Volumes, Ventricular" OR "Fraction, Ventricular Ejection" OR "Fractions, Ventricular Ejection" OR "Stroke Volumes" OR "Ventricular Ejection Fraction" OR "Ventricular Ejection Fractions" OR "Ventricular End Diastolic Volume" OR "Ventricular End Systolic Volume" OR "Ventricular End-Diastolic Volume" OR "Ventricular End-Diastolic Volumes" OR "Ventricular End-Systolic Volume" OR "Ventricular End-Systolic Volumes" OR "Volume, Stroke" OR "Volume, Ventricular End-Diastolic" OR "Volume, Ventricular End-Systolic" OR "Volumes, Stroke" OR "Volumes, Ventricular End-Diastolic" OR "Volumes, Ventricular End-Systolic" OR "Volumen Sistólico OR Fracción de Eyección Ventricular" OR "Volumen Endodiastolico Ventricular" OR "Volumen Sistólico Final Ventricular" OR "Volume Sistólico OR Fração de Ejeção Ventricular" OR "Volume Ventricular Diastólico Final" OR "Volume Ventricular Sistólico Final" OR "Débit systolique" OR "FEV (Fraction d'Éjection Ventriculaire)" OR "Fraction d'éjection ventriculaire" OR "VES (Volume d'Éjection Systolique)" OR "Volume d'éjection systolique" OR "Volume télédiastolique" OR "Volume télésystolique" OR "Volume ventriculaire en fin de systole" OR "Ventricular Remodeling" OR "Cardiac Remodeling, Ventricular" OR "Cardiac Remodelings, Ventricular" OR "Left Ventricle Remodeling" OR "Left Ventricle Remodelings" OR "Left Ventricular Remodeling" OR "Left Ventricular Remodelings" OR "Myocardial Remodeling, Ventricular" OR "Myocardial Remodelings, Ventricular" OR "Remodeling, Left Ventricle" OR "Remodeling, Left Ventricular" OR "Remodeling, Ventricle" OR "Remodeling, Ventricular" OR "Remodeling, Ventricular Cardiac" OR "Remodeling, Ventricular Myocardial" OR "Remodelings, Left Ventricle" OR "Remodelings, Left Ventricular" OR "Remodelings, Ventricle" OR "Remodelings, Ventricular" OR "Remodelings, Ventricular Cardiac" OR "Remodelings, Ventricular Myocardial" OR "Ventricle Remodeling" OR "Ventricle Remodeling, Left" OR "Ventricle Remodelings" OR "Ventricle Remodelings, Left" OR "Ventricular Cardiac Remodeling" OR "Ventricular Cardiac Remodelings" OR "Ventricular Myocardial Remodeling" OR "Ventricular Myocardial Remodelings" OR "Ventricular Remodeling, Left" OR "Ventricular Remodelings" OR "Ventricular Remodelings, Left" OR "Remodelación Ventricular" OR "Remodelación Cardíaca Ventricular" OR "Remodelación Miocárdica Ventricular" OR "Remodelación Ventricular Izquierda" OR "Remodelación Ventricular Miocárdica" OR "Remodelação Ventricular" OR "Remodelação Cardíaca Ventricular" OR "Remodelação Miocárdica Ventricular" OR "Remodelação Ventricular Esquerda" OR "Remodelage ventriculaire" OR "Remodelage du ventricule cardiaque" OR "Remodelage ventriculaire gauche" OR "Ventricular Function, Left" OR "Function, Left Ventricular" OR "Functions, Left Ventricular" OR "Left Ventricular Function" OR "Left Ventricular Functions" OR "Ventricular Functions, Left" OR "Función Ventricular Izquierda" OR "Função Ventricular Esquerda" OR "Fonction ventriculaire gauche" OR "Ventricular Pressure" OR" "Intraventricular Pressure" OR "Intraventricular Pressures" OR "Pressure, Intraventricular" OR "Pressure, Ventricular" OR "Pressures, Intraventricular" OR "Pressures, Ventricular" OR "Ventricular Pressures" OR "Presión Ventricular" OR "Pressão Ventricular" OR "Pression ventriculaire" OR "Pression intra-ventriculaire" OR "Pression intraventriculaire")	
Embase	('aortic valve disease' OR 'aortic valve diseases' OR 'valve disease, aortic' OR 'aortic valvular heart disease' OR 'aortic valve disorder' OR 'aortic valve disorders' OR 'valve disorder, aortic' OR 'aortic valvular heart disorder' OR 'aortic heart disease' OR 'aortic heart diseases' OR 'heart disease, aortic' OR 'aortic valve stenoses' OR 'stenoses, aortic valve' OR 'stenosis, aortic valve' OR 'valve stenoses, aortic' OR 'valve stenosis, aortic' OR 'aortic stenosis' OR 'stenoses, aortic' OR 'stenosis, aortic')	272
	('tavr' OR 'transcatheter aortic valve implantation' OR 'implantation, heart valve prosthesis' OR 'surgical procedure, cardiac' OR 'surgical procedures, cardiac' OR 'heart surgical procedure' OR 'heart surgical procedures' OR 'procedure, heart surgical' OR 'procedures, heart surgical' OR 'surgical procedure, heart' OR 'surgical procedures, heart' OR 'cardiac surgical procedure' OR 'procedures, cardiac surgical' OR 'procedure, cardiac surgical')	
	('ventricular function, left' OR 'left ventricular function' OR 'function, left ventricular' OR 'functions, left ventricular' OR 'left ventricular functions' OR 'ventricular functions, left' OR 'ventricular remodeling' OR 'remodelings, ventricular' OR 'remodeling, ventricular' OR 'ventricular remodelings' OR 'cardiac remodeling, ventricular' OR 'cardiac remodelings, ventricular' OR 'remodelings, ventricular cardiac' OR 'remodeling, ventricular cardiac' OR 'ventricular cardiac remodeling' OR 'ventricular cardiac remodelings' OR 'myocardial remodeling, ventricular' OR 'myocardial remodelings, ventricular' OR 'remodelings, ventricular myocardial' OR 'remodeling, ventricular myocardial' OR 'ventricular myocardial remodeling' OR 'ventricular myocardial remodelings' OR 'ventricle remodeling' OR 'remodelings, ventricle' OR 'remodeling, ventricle' OR 'ventricle remodelings' OR 'left ventricle remodeling' OR 'left ventricle remodelings' OR 'remodeling, left ventricle' OR 'remodelings, left ventricle' OR 'ventricle remodeling, left' OR 'ventricle remodelings, left' OR 'left ventricular remodeling' OR 'left ventricular remodelings' OR 'remodeling, left ventricular' OR 'remodelings, left ventricular' OR 'ventricular remodeling, left' OR 'ventricular remodelings, left' OR 'ventricular pressure' OR 'pressures, ventricular' OR 'pressure, ventricular' OR 'ventricular pressures' OR 'intraventricular pressure' OR 'intraventricular pressures' OR 'pressure, intraventricular' OR 'pressures, intraventricular' OR 'stroke volumes' OR 'volumes, stroke' OR 'volume, stroke' OR 'ventricular ejection fraction' OR 'ejection fractions, ventricular' OR 'ejection fraction, ventricular' OR 'fractions, ventricular ejection' OR 'fraction, ventricular ejection' OR 'ventricular ejection fractions' OR 'ventricular end-diastolic volume' OR 'end-diastolic volumes, ventricular' OR 'end-diastolic volume, ventricular' OR 'ventricular end diastolic volume' OR 'ventricular end-diastolic volumes' OR 'volumes, ventricular end-diastolic' OR 'volume, ventricular end-diastolic' OR 'ventricular end-systolic volume' OR 'end-systolic volumes, ventricular' OR 'end-systolic volume, ventricular' OR 'ventricular end systolic volume' OR 'ventricular end-systolic volumes' OR 'volumes, ventricular end-systolic' OR 'volume, ventricular end-systolic')	
	('randomized controlled trial' OR 'randomised controlled trial' OR 'clinical trials, randomized' OR 'trials, randomized clinical' OR 'controlled clinical trials, randomized' OR 'controlled clinical trial' OR 'clinical trial')	
Total search		1532
Total duplicates before revision of RAYYAN		409
Duplicates		255
Not duplicate		15
Resolved		139
Total registers after duplicates removed		1532 – 255 = 1277

Meta-analyses of outcomes

Meta-Analysis for 30-Day All-Cause Mortality

**Figure 2:**
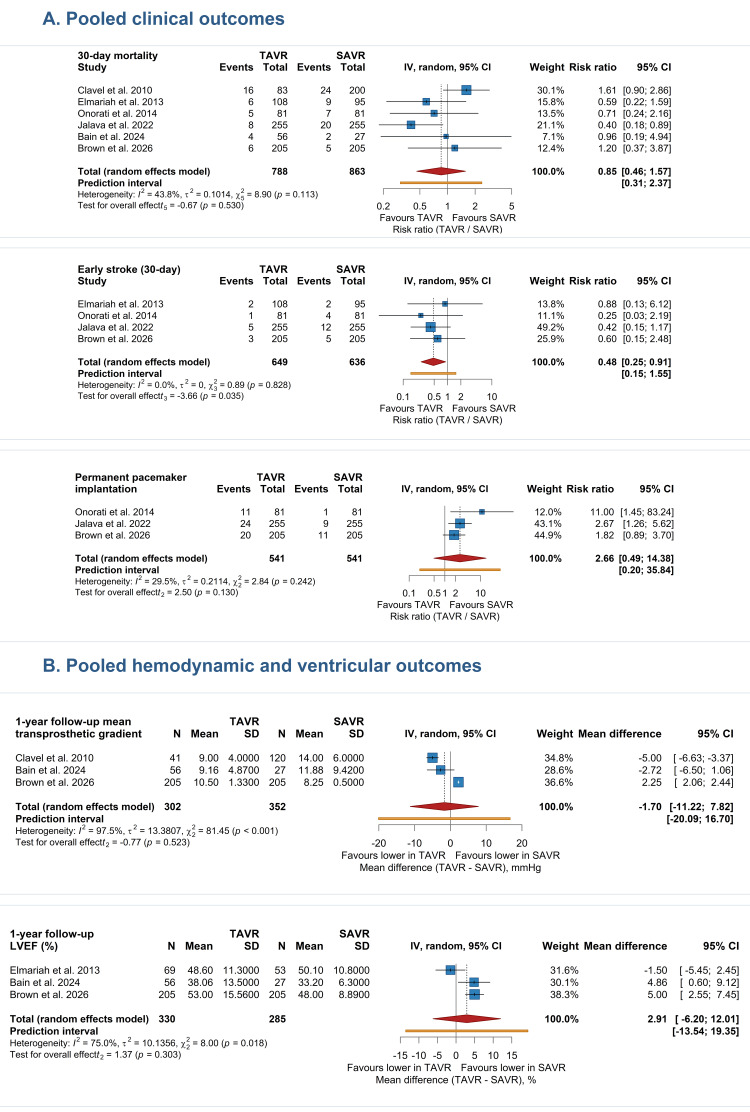
Forest plots for pooled outcomes. Panel A, pooled clinical outcomes (30-day all-cause mortality, early stroke, and permanent pacemaker implantation) [14-16,18-20]; and Panel B, pooled hemodynamic and ventricular outcomes (one-year follow-up mean transprosthetic gradient and one-year follow-up LVEF) [14,15,19,20].

Thirty-day all-cause mortality was reported in six studies, including 1,651 patients [14-16,18-20]. The pooled analysis did not demonstrate a statistically significant difference between TAVR/TAVI and SAVR (RR 0.85, 95% CI 0.46-1.57; I^2^ = 43.8%). This finding is clinically important because reduced LVEF is traditionally associated with higher perioperative vulnerability. However, the result should be interpreted as suggesting broadly similar early survival rather than proof of equivalence, because the confidence interval remains wide and certainty of evidence was low (Figure 2).

Meta-Analysis for Early Stroke (30-Day)

Early stroke was reported in four studies, including 1,285 patients [15,16,18,20]. TAVR was associated with a significantly lower pooled risk of early stroke compared with SAVR (RR 0.48, 95% CI 0.25-0.91; I^2^ = 0.0%). Although the absolute number of events was small, the direction of effect was consistent across the pooled estimate (Figure 2).

Meta-Analysis for Permanent Pacemaker Implantation

Permanent pacemaker implantation was reported in three studies, including 1,082 patients [16,18,20]. The pooled estimate numerically favored SAVR, with a higher rate after TAVR, but the confidence interval was wide and crossed the null (RR 2.66, 95% CI 0.49-14.38; I²=29.5%). Accordingly, this finding was interpreted as imprecise despite the directional signal toward more conduction-related device implantation after TAVR (Figure 2).

Meta-Analysis for One-Year Follow-up Mean Transprosthetic Gradient

Three studies, including 654 patients, reported the one-year or closest follow-up mean transprosthetic gradient [14,19,20]. No significant between-group difference was observed (MD −1.70 mmHg, 95% CI −11.22 to 7.82). Heterogeneity was extreme (I²=97.5%), indicating marked variation in the magnitude and direction of study-level effects and limiting the certainty of the pooled estimate (Figure 2).

Meta-Analysis for One-Year Follow-up LVEF

Three studies, including 615 patients, contributed data for one-year follow-up LVEF [15,19,20]. The pooled analysis did not identify a statistically significant between-group difference (MD 2.91 percentage points, 95% CI −6.20 to 12.01; I²=75.0%). Thus, available evidence does not demonstrate a clear advantage of either strategy for one-year ventricular systolic recovery in this subgroup (Figure 2).

Risk of Bias Assessment

Risk-of-bias judgments differed according to study design [10,11]. The two randomized subgroup analyses were judged as having some concerns overall, mainly because of subgroup-specific limitations and concerns related to reporting or deviation domains [15,17]. Among the observational studies, Jalava et al. (2022) [18] and Onorati et al. (2014) [16] were judged at moderate risk of bias overall, whereas Brown et al. (2026) [20], Bain et al. (2024) [19], and Clavel et al. (2010) [14] were judged at serious risk of bias, primarily due to confounding and selection into the analysis. Detailed domain-level judgments are shown in Table 3.

**Table 3:** Risk of bias assessment [10,11]. ROBINS-I domains: D1, bias due to confounding; D2, classification of interventions; D3, selection of participants into the study/analysis; D4, missing data; D5, measurement of outcomes; D6, selection of the reported result [10]. *Randomized studies were assessed with RoB 2: D1, randomization process; D2, deviations from intended interventions; D3, missing outcome data; D4, measurement of outcomes; D5, selection of the reported result [11]. RoB 2: revised Cochrane risk-of-bias tool for randomized trials, ROBINS-I: risk of bias in non-randomized studies of interventions.

Study	D1	D2	D3	D4	D5	D6	Overall
Brown et al., 2026 [20]	Moderate	Low	Serious	Low	Low	Low	Serious
Bain et al., 2024 [19]	Serious	Low	Low	Low	Low	Low	Serious
Jalava et al., 2022 [18]	Moderate	Low	Low	Low	Low	Low	Moderate
Onorati et al., 2014 [16]	Moderate	Low	Moderate	Low	Low	Low	Moderate
Clavel et al., 2010 [14]	Serious	Low	Moderate	Low	Low	Low	Serious
Ito et al., 2020* [17]	Low	Low	Low	Low	Some concerns	—	Some concerns
Elmariah et al., 2013* [15]	Low	Some concerns	Low	Low	Some concerns	—	Some concerns

Certainty of the Evidence

Certainty of evidence was assessed using the GRADE approach and is summarized in Table 4; reasons for downgrading are presented in Table 5.

**Table 4:** GRADE certainty assessment of key outcomes [12,13]. GRADE, Grading of Recommendations Assessment, Development and Evaluation; LVEF, left ventricular ejection fraction; RR, risk ratio; MD, mean difference.

Outcome	Participants (studies)	Assumed risk with SAVR	Corresponding risk with TAVR	Relative effect (95% CI)	Certainty of evidence
30-day all-cause mortality	1,651 (6)	78 per 1,000	66 per 1,000 (36 to 122)	RR 0.85 (0.46 to 1.57)	Low
Early stroke (30-day)	1,285 (4)	36 per 1,000	17 per 1,000 (9 to 33)	RR 0.48 (0.25 to 0.91)	Low
Permanent pacemaker implantation	1,082 (3)	39 per 1,000	103 per 1,000 (19 to 558)	RR 2.66 (0.49 to 14.38)	Very low
1-year follow-up mean transprosthetic gradient	654 (3)	Mean in SAVR group: 10.49 mmHg	1.70 mmHg lower (11.22 lower to 7.82 higher)	MD −1.70 mmHg (−11.22 to 7.82)	Very low
1-year follow-up LVEF	615 (3)	Mean in SAVR group: 46.99%	2.91 percentage points higher (6.20 lower to 12.01 higher)	MD 2.91% (−6.20 to 12.01)	Very low

**Table 5:** Reasons for downgrading certainty across outcomes [12,13]. GRADE, Grading of Recommendations Assessment, Development and Evaluation; LVEF, left ventricular ejection fraction.

Outcome	Risk of bias	Inconsistency	Indirectness	Imprecision	Publication bias
30-day all-cause mortality	Serious	Not serious	Not serious	Serious	Undetected
Early stroke (30-day)	Serious	Not serious	Not serious	Serious	Undetected
Permanent pacemaker implantation	Serious	Not serious	Not serious	Very serious	Undetected
1-year follow-up mean transprosthetic gradient	Serious	Very serious	Not serious	Serious	Undetected
1-year follow-up LVEF	Serious	Serious	Not serious	Serious	Undetected

Discussion

This systematic review and meta-analysis synthesized comparative evidence focused on adults with severe AS and reduced baseline LVEF undergoing TAVR/TAVI or SAVR. Three main findings emerged. First, TAVR/TAVI was associated with a lower pooled risk of early stroke. Second, 30-day all-cause mortality did not show a statistically significant difference between strategies, suggesting broadly similar early survival rather than proven equivalence. Third, available one-year hemodynamic and ventricular recovery outcomes remain very uncertain and do not establish a clear advantage for either approach. This uncertainty is clinically relevant because myocardial fibrosis, follow-up valve hemodynamics, and prosthesis-patient mismatch may influence ventricular recovery and clinical outcomes after valve replacement [21-23].

The absence of a statistically significant difference in 30-day mortality is clinically important. Reduced LVEF has traditionally been viewed as a marker of increased operative vulnerability, particularly in the setting of long-standing pressure overload, low-flow physiology, myocardial fibrosis, and coexisting ischemic disease [6,21]. Within this focused, reduced-LVEF evidence base, short-term survival appeared broadly similar after TAVR/TAVI and SAVR. However, this should not be interpreted as definitive equivalence because confidence intervals were wide, the evidence base was small, and certainty was low.

The pooled signal favoring TAVR/TAVI for early stroke warrants cautious interpretation. A less invasive procedure, avoidance of cardiopulmonary bypass, and a greater representation of transfemoral practice in more contemporary cohorts may partly explain the observed direction of effect. However, the total number of events was small, and the finding was derived from only four studies. Accordingly, this result should be viewed as a potentially relevant signal rather than definitive mechanistic superiority [24].

Permanent pacemaker implantation was numerically more frequent after TAVR/TAVI, but the estimate was highly imprecise. This finding reinforces the importance of procedural and device heterogeneity. Conduction-related complications after TAVR/TAVI may differ by valve platform, valve generation, implantation depth, baseline conduction disease, membranous septum anatomy, annular or LVOT calcification, and operator technique [25-27]. Older SAVR-focused evidence should not be extrapolated directly to contemporary TAVR practice. Because the included studies did not consistently report pacemaker outcomes stratified by transcatheter valve type, valve generation, implantation depth, or access route, the pooled estimate should be interpreted as a broad treatment-strategy-level signal rather than a device-specific estimate applicable to all TAVR/TAVI platforms.

The hemodynamic and ventricular recovery findings were more ambiguous. Mean transprosthetic gradient at one year showed no statistically significant pooled difference, but heterogeneity was extreme. Likewise, one-year LVEF did not differ significantly between treatment strategies, and heterogeneity was substantial. These findings may reflect differences in baseline risk, LVEF thresholds, low-flow status, contractile reserve, myocardial fibrosis burden, valve platforms, access routes, surgical prosthesis types, echocardiographic timing, and study design. Therefore, the results should not be interpreted as proof of equivalence or absence of benefit for either strategy.

The interpretation of mean transprosthetic gradient requires particular caution in reduced-LVEF populations. Doppler gradients are flow-dependent and may vary according to stroke volume, low-flow status, ventricular recovery, prosthesis-patient mismatch, and paravalvular regurgitation [28]. The included studies did not consistently report whether echocardiograms were interpreted by a core laboratory, whether standardized Doppler acquisition protocols were used, whether assessors were blinded to treatment assignment, or whether gradients were adjusted for flow state. Thus, the one-year gradient estimate should be regarded as a study-level comparison of reported echocardiographic gradients rather than a fully standardized, core-laboratory-adjudicated, or flow-adjusted measure of prosthetic valve performance.

Some pooled findings differed from the early report by Clavel et al. [14]. This should be interpreted in the context of study era and baseline risk. Clavel et al. represented an early TAVI-era cohort with a smaller transcatheter sample and higher baseline operative risk in the TAVR/TAVI group compared with the SAVR group. Differences between that study and the pooled estimates may reflect early-generation device technology, procedural-era effects, patient-selection differences, and the inclusion of later randomized subgroup analyses and more contemporary matched cohorts. Thus, the present synthesis does not negate the Clavel et al. findings but contextualizes them within a broader and clinically heterogeneous reduced-LVEF evidence base.

The included evidence spans a broad period from early TAVR experience to contemporary practice. Earlier studies may reflect a pioneer era characterized by older-generation transcatheter valves, less refined implantation techniques, different access-route distributions, and higher baseline operative risk, whereas more recent cohorts may better approximate contemporary practice. Because the available reduced-LVEF evidence was sparse, formal subgroup analyses by study era, valve generation, access route, or surgical technique were not feasible. Consequently, the pooled estimates should not be interpreted as device-generation-specific estimates of contemporary TAVR performance.

The reduced-LVEF population was also physiologically heterogeneous. Included studies used different LVEF thresholds, ranging from LVEF <50% or ≤50% to LVEF ≤35% and one cohort restricted to LVEF ≤25%. These groups may differ in contractile reserve, low-flow physiology, myocardial fibrosis burden, perioperative risk, and likelihood of ventricular recovery. This heterogeneity likely contributed to the inconsistency observed in one-year hemodynamic and ventricular recovery outcomes.

Several limitations should be considered. First, the available evidence base was small and composed predominantly of observational comparative cohorts, making residual confounding and treatment-selection bias important concerns. Baseline risk, frailty, anatomical suitability, porcelain aorta, coronary disease burden, STS-PROM or EuroSCORE, and procedural-era effects may have influenced whether patients underwent TAVR/TAVI or SAVR. Second, the review combined studies from different TAVR eras and reduced-LVEF phenotypes; therefore, findings should not be interpreted as applying uniformly to ultra-low LVEF, mild-to-moderate systolic dysfunction, high-gradient AS, or low-flow, low-gradient AS. Third, severe AS was accepted according to study-level diagnostic criteria, and the review could not reliably stratify patients by true-severe versus pseudo-severe AS, flow-gradient phenotype, or contractile reserve. Fourth, procedural heterogeneity was substantial: TAVR/TAVI device platform, valve generation, access route, implantation technique, surgical approach, and prosthesis type were not consistently reported and could not be explored formally. Fifth, outcome reporting was fragmented, and important outcomes such as cardiovascular mortality, heart failure rehospitalization, and composite heart failure endpoints were not consistently extractable for the reduced-LVEF subgroup. Sixth, echocardiographic quality and flow-adjusted interpretation of gradients were not consistently reported. Finally, several pooled outcomes were affected by imprecision and, for continuous outcomes, substantial inconsistency, which is reflected in the low or very low GRADE certainty ratings.

Despite these limitations, the review addresses a clinically important and understudied subgroup in which therapeutic decisions often require balancing early procedural safety against residual valve performance, conduction-related complications, and myocardial recovery. Future comparative studies should report outcomes stratified by LVEF severity, low-flow status, contractile reserve, myocardial fibrosis, valve platform, valve generation, access route, surgical technique, prosthesis type, and echocardiographic quality-control procedures. Longer follow-up and consistent reporting of cardiovascular mortality, heart failure hospitalization, and patient-centered outcomes are also needed to improve the precision and clinical usefulness of the evidence.

## Conclusions

In adults with severe AS and reduced baseline LVEF, TAVR/TAVI may be associated with a lower risk of early stroke, while short-term all-cause mortality appears broadly similar to SAVR. However, the available evidence is insufficient to establish equivalence or clinically meaningful differences in permanent pacemaker implantation, one-year mean transprosthetic gradient, or one-year LVEF recovery. These outcomes remain limited by small numbers of contributing studies, imprecision, inconsistency, clinical and procedural heterogeneity, and low or very low certainty of evidence. The findings should be used to inform Heart Team discussions at the level of broad treatment-strategy selection, but they should not be interpreted as device-specific, access-route-specific, or LVEF-stratum-specific comparative estimates.
